# Use of Octreotide in Chylothorax With Mediastinal Mass of Unknown Behaviour: A Case Report

**DOI:** 10.1002/rcr2.70471

**Published:** 2026-01-15

**Authors:** Arnold Joseph Geronimo, Manuel Monis

**Affiliations:** ^1^ Department of Internal Medicine Ilocos Training and Regional Medical Center San Fernando City Philippines

**Keywords:** chylothorax, mediastinal mass, octreotide

## Abstract

Chylothorax poses a clinical challenge, especially in resource‐limited settings where diagnosis and treatment require innovation. This report details the successful use of octreotide in a 37‐year‐old female with non‐traumatic chylothorax secondary to a mediastinal mass. She presented with chylothorax symptoms, and imaging showed a mediastinal mass, but tissue biopsy was refused, and advanced interventions were unavailable. Octreotide infusion reduced chest tube output and led to the complete resolution of chylothorax. This case demonstrates that octreotide can provide effective symptom control and clinical stabilisation in mediastinal mass‐associated chylothorax when surgical or advanced options are not feasible, serving as a bridge for further workup and treatment planning.

## Graphical abstract

A 37‐year‐old woman with chylothorax and a mediastinal mass of uncertain behaviour had persistent pleural drainage despite initial interventions and unavailable advanced options. Octreotide infusion resulted in a marked reduction of drainage and complete resolution. This case highlights the successful symptom control achieved with octreotide as a non‐surgical intervention in resource‐limited settings, providing time for further diagnostics and treatment planning.

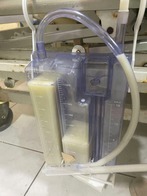

## 1 Introduction

Chylothorax, the build‐up of chyle in the pleural space from thoracic duct disruption, is difficult to manage due to many causes, such as trauma, malignancy and idiopathic cases. Determining the cause is key for effective treatment and patient outcomes, but in resource‐limited areas, a lack of advanced diagnostics requires adaptable strategies. Octreotide, a synthetic somatostatin, may help manage non‐traumatic chylothorax, though ideal dosing is uncertain. This report reviews the use of octreotide in a patient with a mediastinal mass who declined biopsy, offering insights for clinical care in challenging situations.

## 2 Case Report

The patient is a 37‐year‐old Filipino female who was referred to the Ilocos Training and Regional Medical Center due to a two‐week history of productive cough, dyspnea, generalised weakness and anorexia. She has no known comorbidities, an unremarkable family history, and is a non‐smoker. Physical examination revealed coarse crackles over the left lung and diminished breath sounds on the right.

**FIGURE 1:**
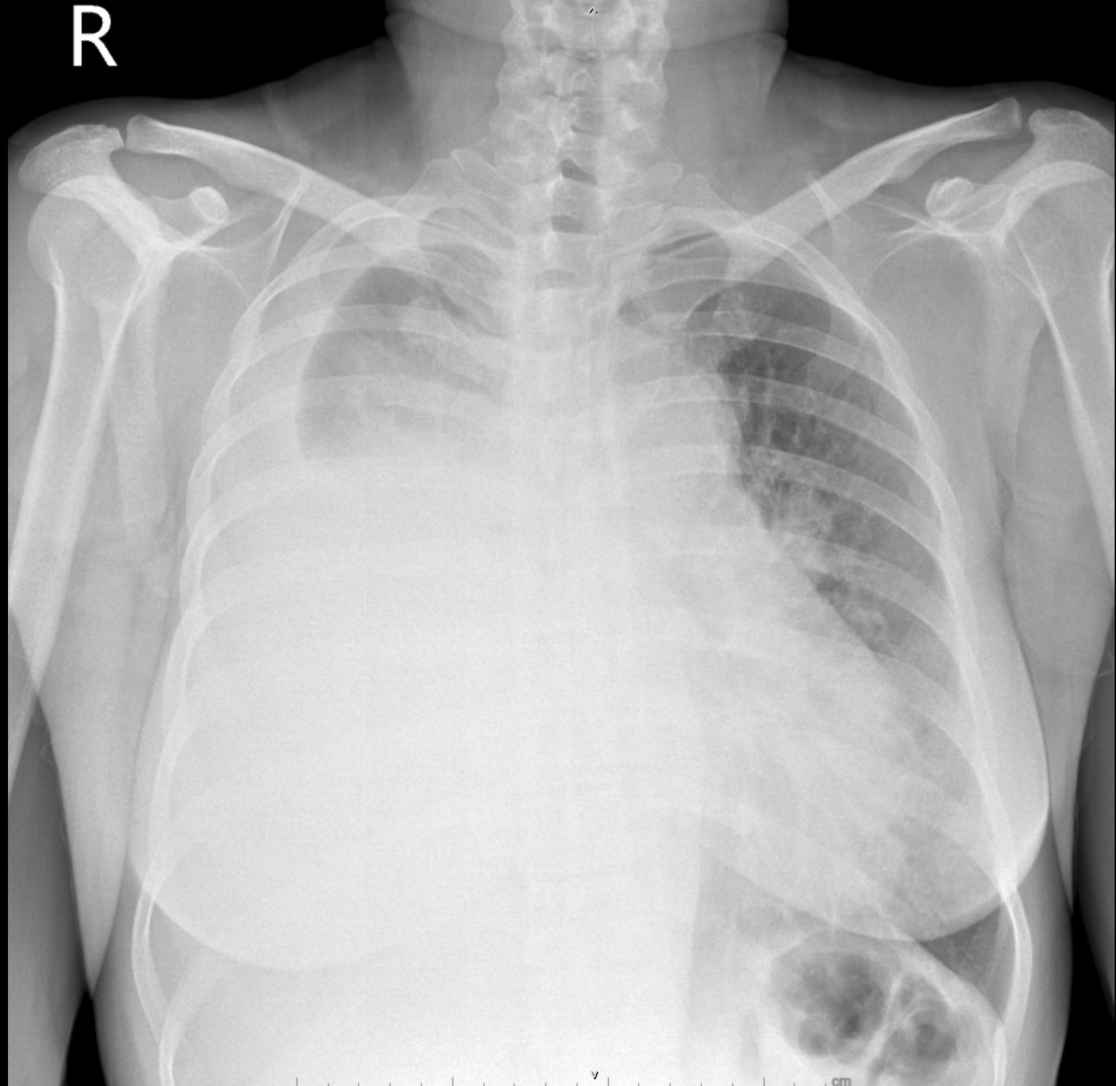
Chest X‐ray PA showing right‐sided hydrothorax with noted soft tissue density in the right parahilar area, which may relate to a mediastinal mass.

**FIGURE 2:**
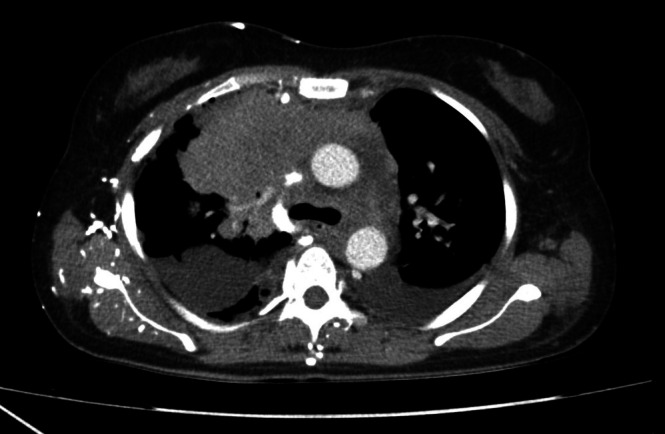
Chest CT scan revealing right upper lobe mass extending to the superoanterior mediastinum.

Chest radiography revealed a massive right‐sided pleural effusion (Figure 1), quantified at approximately 1900 mL via chest ultrasound, alongside a soft tissue density in the right parahilar region, suggestive of a possible mediastinal mass. Contrast‐enhanced CT imaging demonstrated a lobulated, isodense, heterogeneously enhancing mass in the anterior segment of the right upper lobe extending to the right anterosuperior mediastinal compartment (Figure 2).

**FIGURE 3:**
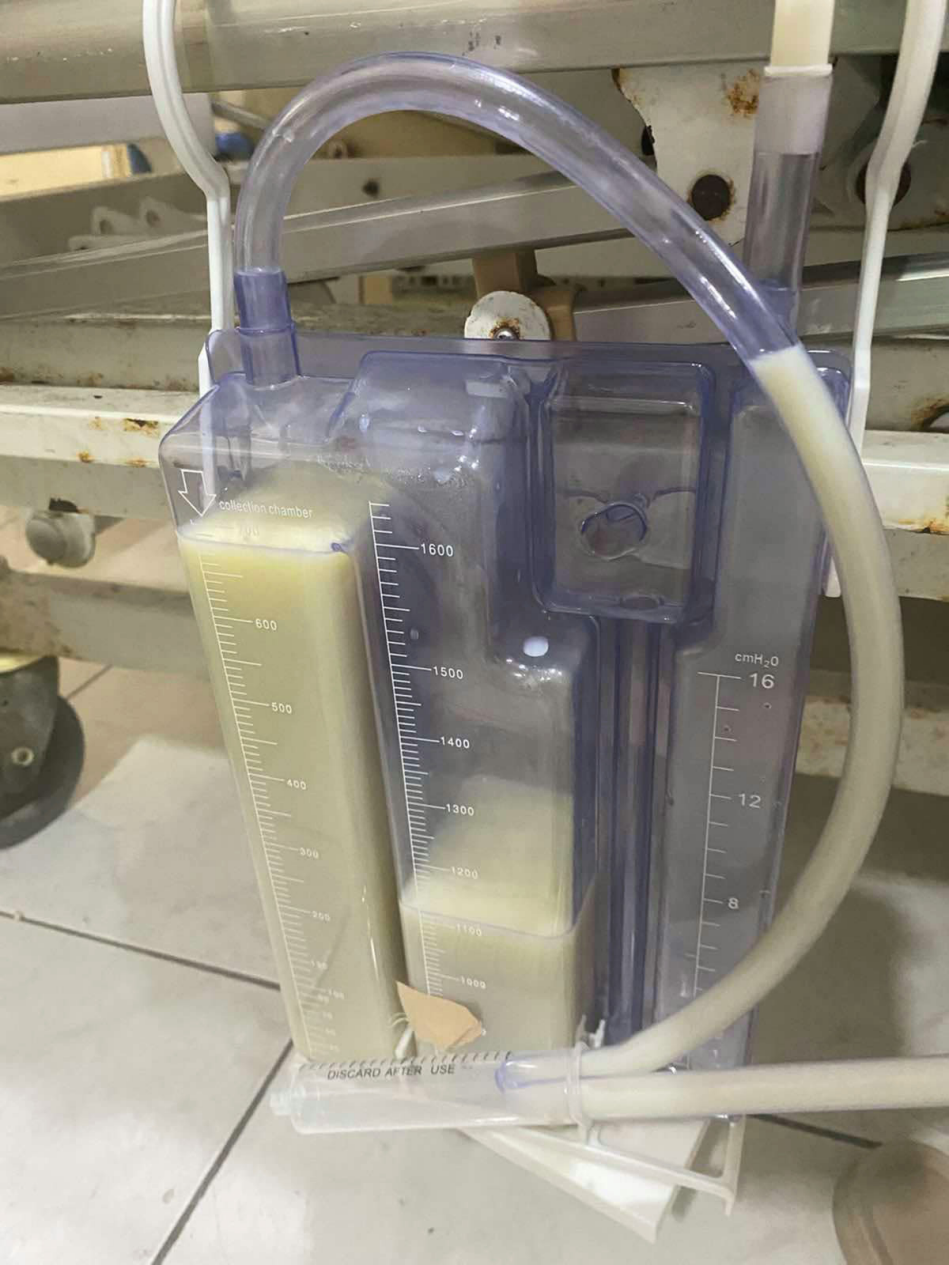
Pleural effusion with milky chest tube output.

A chest tube was placed, draining a milky chylous effusion with triglycerides of 359 mg/dL, cholesterol of 40 mg/dL, LDH of 302 U/L and protein of 23.16 g/L, with a daily output of 250–500 mL (Figure 3). Despite recommendations for a biopsy of the mediastinal mass, the patient declined. Serum levels of B‐HCG, AFP and LDH were within normal limits.

The case was referred to a medical nutritionist who recommended a low‐fat diet supplemented with medium‐chain triglycerides. The chest tube continued to drain 250–500 mL daily. Due to the unavailability of lymphoscintigraphy, thoracic duct ligation and radiation therapy, the patient was managed medically. Octreotide infusion was initiated at 0.1 mg (1 mL of 0.1 mg/mL solution) in 100 mL of normal saline, administered continuously for 16 h a day. On the ninth infusion day, chest tube output decreased to 160 mL over 24 h. By the second week of treatment, chest tube output had further declined, reaching as low as 5 mL/24 h, and the patient was discharged with a Heimlich valve. A follow‐up visit 1 week later showed no output, and the Heimlich valve was removed. The patient was advised to proceed with a tumour biopsy and consented to the plan.

## 3 Discussion

Chylothorax results from the disruption of the thoracic duct or its branches, causing the leakage of chyle, a lymphatic fluid derived from the intestines, into the pleural cavity [1]. This can occur due to trauma or non‐traumatic causes. The initial step in diagnosing chylothorax typically involves testing the cholesterol and triglyceride levels of pleural fluid. A triglyceride concentration greater than 1.24 mmol/L (> 110 mg/dL) and a cholesterol level below 5.18 mmol/L (< 200 mg/dL) confirm the diagnosis [2]. A triglyceride level above 1.24 mmol/L strongly suggests chylothorax, with only a 1% likelihood of non‐chylous disease. Conversely, a triglyceride level below 0.56 mmol/L (< 50 mg/dL) carries a 5% risk of chylous effusion [2].

The primary management of malignant chylothorax involves therapeutic thoracentesis to relieve respiratory distress. However, prolonged chest tube drainage is associated with complications such as infections and nutritional depletion, making it unsuitable for long‐term management [3]. In non‐traumatic chylothorax, addressing the underlying cause is typically the approach, but its success depends on the type and severity of the underlying condition. Treatment options include surgical interventions such as thoracic duct ligation, pleurodesis, thoracic duct embolization, as well as chemotherapy and radiation [4, 5]. However, patients with advanced malignancies may continue to deteriorate despite these treatments and may be too fragile to undergo surgery.

Octreotide, a synthetic somatostatin analog, has shown promise in managing chylothorax by reducing splanchnic blood flow and inhibiting lymphatic secretion. This helps control chyle leakage and offers anti‐inflammatory benefits, contributing to the resolution of pleural effusion and alleviating respiratory distress. The optimal dosing and administration routes remain unclear; some cases utilised a starting dose of 50 mcg every 8 h [3].

In resource‐limited settings, where access to specialised care may be limited, octreotide is crucial in managing chylothorax, especially in cases involving a mediastinal mass of uncertain aetiology. This is the first reported case in the Philippines documenting the use of octreotide in the context of a mediastinal mass of unknown behaviour.

When a mediastinal mass with unknown behaviour is present, octreotide becomes especially relevant. While definitive treatment for the mass may require surgery or targeted therapies, octreotide provides symptomatic relief and stabilisation, particularly when immediate surgical resection is not feasible. By reducing chyle production and managing pleural effusion, octreotide is a valuable temporising measure while diagnostic evaluation and treatment planning for the mediastinal mass continue.

## Author Contributions

All authors listed contribute equally to conceptualisation, text drafting and manuscript review.

## Consent

The authors declare that written informed consent was obtained for the publication of this manuscript and accompanying images using the consent form provided by the Journal.

## Conflicts of Interest

The authors declare no conflicts of interest.

## Data Availability

Data sharing not applicable to this article as no datasets were generated or analysed during the current study.
